# Neighborhood Environment and Mental Well-Being Among Chinese Older Adults: The Mediating Role of Social Capital

**DOI:** 10.1093/geroni/igac070

**Published:** 2022-11-17

**Authors:** Shiyu Lu, Yingqi Guo, Cheryl Chui, Yuqi Liu, On Fung Chan, Samuel W Chan, Terry Y S Lum

**Affiliations:** Department of Social and Behavioral Sciences, City University of Hong Kong, Hong Kong, China; Department of Social Work; Department of Geography; Smart Society Lab, Hong Kong Baptist University, Hong Kong, China; Department of Social Work and Social Administration, The University of Hong Kong, Hong Kong, China; Department of Urban Planning, South China University of Technology, Guangzhou, China; Department of Social Work and Social Administration, The University of Hong Kong, Hong Kong, China; Department of Social Work and Social Administration, The University of Hong Kong, Hong Kong, China; Department of Social Work and Social Administration, The University of Hong Kong, Hong Kong, China

**Keywords:** Age-friendly environment, Mental health, Quality of life, Social networks

## Abstract

**Background and Objectives:**

Neighborhood environments are increasingly recognized as associated with mental well-being among older adults. However, their underlying mechanisms remain unclear. This study investigated mediating effects of cognitive and structural social capital (SC) in relationships between neighborhood environments and mental well-being among older adults.

**Research Design and Methods:**

We conducted a cross-sectional analysis of 1,277 community-dwellers aged 60 years and older in Hong Kong in 2021. The Warwick–Edinburgh Mental Well-being Scale assessed mental well-being. Perceived age-friendly environment was assessed. Objective neighborhood environment was measured by the number of neighborhood facilities (e.g., transportation, community centers, leisure facilities) within 200-m and 500-m buffer areas from respondents’ residences. Structural equational modeling was used.

**Results:**

Perceived age-friendly environment regarding community and health support had a protective role on mental well-being. More community centers were directly associated with better affective-emotional well-being, while more passive leisure facilities directly lowered psychological-functioning well-being. Cognitive SC outweighed structural SC in mediating relationships of neighborhood environment on mental well-being.

**Discussion and Implications:**

Our findings advance the ecological model of aging by providing evidence for cognitive and structural SC as mediators to explain the relationship between neighborhood environment and mental well-being. Policy implications for optimizing mental well-being in aging societies are discussed.

**Translational Significance:** The mechanisms of how neighborhood environments influence older adults’ mental well-being remain unclear. The study found that perceived age-friendly environment regarding community and health support had a protective role on mental well-being. More community centers led to better affective-emotional well-being, while more passive leisure facilities lowered psychological-functioning well-being. Cognitive social capital outweighed structural ones in mediating relationships of neighborhood environment on mental well-being. Ensuring more community centers are available for older adults and tailoring the parks and public transportation to older adults’ needs/preferences are beneficial for their well-being.

Mental well-being includes the presence of hedonic and eudaimonic well-being (Clarke et al., 2011; Ryan & Deci, 2001). Hedonic well-being refers to the subjective experience of happiness and life satisfaction, while eudaimonic well-being focuses on positive psychological functioning, including autonomy and self-realization (Ryan & Deci, 2001). Mental well-being plays a vital role in later life and is associated with reduced risks of morbidity and mortality (Steptoe et al., 2014; Trudel-Fitzgerald et al., 2021). Literature has shown a range of determinants of old adults’ mental well-being, including but not limited to age, gender, marital status, and socioeconomic status (Mavali et al., 2020). Many of these individual-level factors are difficult to modify in old age.

Social capital (SC) has emerged as a modifiable factor for promoting older adults’ mental well-being. There is no consensus on how to define SC. One of the widely used definitions is “features of social organization, such as trust, norms, and networks that can improve the efficiency of society by facilitating coordinated actions” (Putnam, 1993, p. 3). SC is presented in many forms but can be categorized into structural and cognitive dimensions. Structural SC refers to externally observable social interactions between people and groups/organizations, whereas the cognitive dimension consists of people’s norms, values, and beliefs that affect their participation in society (Moore & Kawachi, 2017). SC is associated with better mental health among older adults (Rodgers et al., 2019). Systematic reviews reveal that the evidence for protective effects of cognitive SC on mental health is stronger than the structural dimension (Ehsan et al., 2019). From a life-course perspective, older adults are more likely to experience shrinking social networks in their community due to mobility decline and the loss of social partners (Wrzus et al., 2013). It is imperative to optimize opportunities to facilitate SC and mental well-being in later life.

Increasing attention has been paid to understanding how neighborhood design suppresses and enhances opportunities for improving older adults’ SC and mental health (Mouratidis, 2019). The ecology theory of aging (ETA) posits that neighborhood environments profoundly influence older adults as they encounter varying levels of functional limitations and spend more time within their communities (Lawton, 1977; Lawton & Nahemow, 1973). WHO emphasized the importance of building age-friendly neighborhoods to optimize opportunities for promoting mental well-being as people age (World Health Organization, 2015).

Nevertheless, most studies exploring how neighborhood environment influences older adults’ mental well-being rely on subjective measures of neighborhood environment (Padeiro et al., 2022). Few incorporate both subjective and objective attributes of neighborhood environments. The subjective attributes of neighborhood environments involve a psychological evaluation of whether the surroundings match personal capacities and needs (Guo et al., 2021). Given the importance of an age-friendly environment, an increasing number of studies report positive but small effects of perceived age-friendly environments on older people’s life satisfaction and quality of life (Gibney et al., 2020; Nieboer & Cramm, 2018). Some studies have reported positive effects of specific domains of age-friendly environments, like community support and health services, social participation, outdoor space and buildings for older adults’ mental well-being (Guo et al., 2021). Conversely, objective attributes of neighborhood environments can be captured using Geographical Information Systems (Park et al., 2021). The advantages of using objective attributes of neighborhood environment involve the reduction of recall and same-source bias (Hassan, 2006). Special attention has been paid to how the availability of neighborhood facilities (e.g., recreational, transport, parks) affects older people’s mental health as these facilities could satisfy older people’s daily needs for social interaction, and they could be well planned (Guo et al., 2021). However, the evidence linking objective attributes of neighborhood environment and older people’s mental well-being is far from established. Evidence that higher provision of neighborhood facilities is associated with better mental well-being among older adults has been generated mainly from the West (Peace et al., 2005). The adverse effects of the larger number of parks and transportation on older adults’ mental health were found in Asia (Lu et al., 2021). Such mixed evidence may reflect that the impact of neighborhood facilities on older adults’ well-being is complicated and depends on the contexts where older adults live. For instance, compared to Western cities with relatively low density, Hong Kong is high-density with scattered urban greenness; the available parks near the neighborhood may be small and cannot meet older adults’ daily leisure use (Lu et al., 2021). Hence, mixed evidence related to the impacts of neighborhood facilities on older adults’ well-being deserves further investigation.

More importantly, theoretical understanding of how neighborhood environment, SC, and mental well-being are meaningfully linked among older adults remains limited. To better understand pathways in relationships between subjective and objective attributes of neighborhood environment and mental well-being among older adults, the current study aimed to test cognitive and structural SC as mediators.

## Theoretical Framework

According to ETA (Lawton & Nahemow, 1973), neighborhood environment can impose constraints or enhance opportunities for maintaining mental well-being in late life, depending on whether there is a good fit between personal competence (e.g., cognitive level) and neighborhood environmental characteristics (e.g., barrier-free neighborhood). Two processes of the person–environment fit can be further identified. The experience-driven process involves the development of a sense of positive connection (e.g., belonging) with other people and the environment. The behavior-driven process theorizes that neighborhood environments can promote and stimulate older adults’ proactive behaviors (e.g., physical activities, building social networks) to achieve better mental well-being (Wahl et al., 2012). Riding on the ETA and SC theory, we propose a theoretical framework to examine pathways in relationships between neighborhood environment and mental well-being in later life, by conceptualizing cognitive and structural SC as two parallel mediators in the “meaning-based” and “network-based” processes.

The meaning-based process emphasizes that neighborhood environments create belonging, trust, and reciprocity, leading to better mental well-being. Theories about place attachment emphasize the transformation of “space” into “place” and the emotional bonding between individuals and their living place (Giuliani, 2003) and describe how people form social bonds to neighborhood environments and their neighbors (Scannell & Gifford, 2010). The meaning-based pathway can further link environment-related experiences into cognitive SC formation. Previous studies found that neighborhood environments with available community facilities can enhance older adults’ sense of community, leading to better mental well-being (Guo et al., 2021). In contrast, the network-based process emphasizes neighborhood environments can facilitate structural SC, leading to better mental well-being. The network-based process highlights essential functions of neighborhood environment, including stimulation (e.g., encouraging new forms of social participation) and support (supporting social contact living in barrier-free neighborhoods). Previous studies found that age-friendly neighborhoods with walkable design can facilitate social encounters (Barnett et al., 2017). Some studies have revealed that the availability of neighborhood facilities enables social participation (Levasseur et al., 2011; Lu et al., 2021).

**Figure 1.**
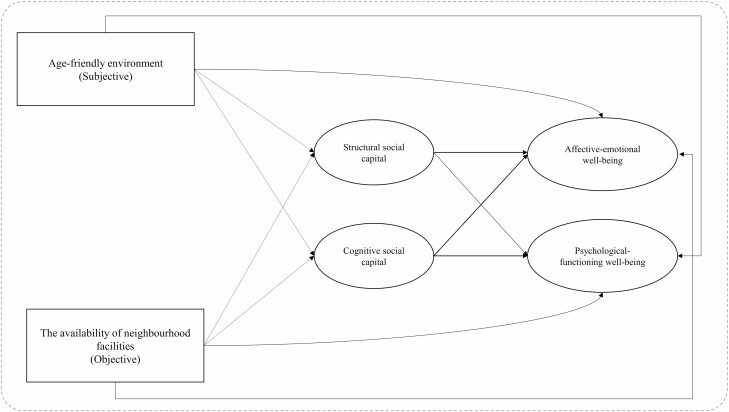
Theoretical framework.

In sum, although relationships between neighborhood environment and SC can be conceptualized through two separate pathways, few have linked SC theory to the ETA and investigated these two pathways simultaneously in relationships between neighborhood environment and mental well-being in old age. Understanding pathways between neighborhood environment and mental well-being may provide an opportunity to develop policy interventions to optimize mental well-being for aging societies. This study addresses the following research questions: (1) how are neighborhood environments associated with mental well-being in later life? (2) Do meaning-based and network-based processes mediate effects of neighborhood environment on late-life mental well-being? We included the perceived age-friendly environment (subjective attributes) and the availability of neighborhood facilities (objective attributes) that are policy-relevant. We conceptualized cognitive and structural SC as mediators. We formulated following hypotheses: (H1) perceived age-friendly environment and the availability of neighborhood facilities are associated with better mental well-being among older adults; (H2) cognitive and structural SC mediate effects of perceived age-friendly environment and the availability of neighborhood facilities on mental well-being. The conceptual framework is presented in Figure 1.

## Research Methods

### Setting

Hong Kong is undergoing rapid population aging: the percentage of the population aged 65 and older is projected to double from 18% in 2019 to 35% in 2069 (HK Census and Statistics Department, 2020). As a high-density city, Hong Kong presents unique environmental features, like an efficient public transportation system, which may influence older adults’ opportunities for maintaining their structural and cognitive SC and mental well-being.

### Sampling and Procedure

This study used purposive and snowball sampling to recruit participants. Participants were eligible if they were aged 18 or older and able to understand and communicate in Cantonese. Data were collected between June and October 2021 through telephone interviews by trained research assistants. This study was approved by the Human Research Ethics Committee of the University of Hong Kong. All participants provided informed consent. To align with our research objectives, we included 1,304 respondents aged 60 years old out of 1,700 respondents aged 18 and older. Of these, 97.9% provided their home address. The final sample for data analysis was 1,277.

### Measurements

*Mental well-being* was assessed by the seven-item short Warwick–Edinburgh Mental Well-being Scale (SWEMWBS; Clarke et al., 2011). SWEMWBS broadly assesses affective-emotional dimensions and psychological functioning. The SWEMWBS uses a 5-point Likert scale (1 = none of the time to 5 = all of the time). Total scores range from 7 to 35, with higher scores indicating better mental well-being. We used the validated Chinese version of the SWEMWBS scale, which has good reliability and validity (Sun et al., 2019). In our sample, Cronbach’s α of SWEMWBS was 0.77.

#### Social capital

***Cognitive SC*** was evaluated by three dimensions: trust, reciprocity, and a sense of belonging (De Silva et al., 2006). *Trust* was measured by the six-item Generalized Trust Scale (GTS; Yamagishi & Yamagishi, 1994) based on a 5-point Likert scale (1 = completely disagree to 5 = completely agree). A higher score indicates a higher level of trust. As there is no Chinese version of GTS, this scale was translated into Chinese and then back-translated by bilingual researchers before use. The scale had excellent internal consistency (Cronbach’s α = 0.91) in our sample. *Reciprocity* was measured by two items adapted from De Silva et al. (2006) based on a 5-point scale (1 = completely disagree to 5 = completely agree), such as “Do you think residents are concerned about issues that not only relate to themselves, but also others?” A higher score indicates higher levels of reciprocity. *Sense of belonging* was measured by the eight-item Brief Sense of Community Scale (Peterson et al., 2008) based on a 5-point Likert scale (1 = strongly disagree to 5 = strongly agree). A higher score indicates a greater sense of community. The scale had satisfactory internal consistency (Cronbach’s α = 0.88) in our sample.

**Structural SC** was evaluated by three dimensions, including organizational membership, volunteering, and social participation (De Silva et al., 2006). Respondents were asked if they held *organizational membership* (0 = No, 1 = Yes) in a list of seven categorizations of organizations based on previous studies (De Silva et al., 2006) and culturally adapted by researchers for application in Hong Kong. A total score ranges from 0 to 7, and a higher score indicates a greater number of organizational memberships. The respondents were also asked whether they participated in *volunteering activities* (0 = No, 1 = Yes) and whether they participated in *social activities/services* in the past 3 months (0 = No, 1 = Yes) (Lu et al., 2020). Supplementary Table 1 documents each measurement to construct cognitive and structural SC in detail.

#### Subjective attributes of neighborhood environment

We assessed subjective attributes of neighborhood environment using perceived age-friendless of neighborhood environment to align with the concepts of age-friendly cities proposed by WHO (World Health Organization, 2015). Eight items were derived from the age-friendly cities framework used in previous studies (Chui et al., 2022). The scale comprises eight domains of age-friendliness: outdoor space and buildings, transportation, housing, social participation, respect, civic participation, communication and information, and community support and health services. Respondents were asked to rate how they perceived their community’s age-friendliness on each domain from a 6-point Likert scale (1 = strongly disagree to 6 = strongly agree). A higher score reflects a higher level of perceived age-friendliness in the corresponding domains.

#### Objective attributes of neighborhood environment

We geocoded respondents’ residential addresses and used road-network-based service area buffers adjusted by terrain and slope from each building block to define the scope of neighborhoods to measure the objective indicators of neighborhood environment. ArcGIS version 10.8.2 was used to perform geocoding and spatial buffering analysis. We created the 200-m and 500-m buffers to extract the number of neighborhood facilities. The 500-m buffer was selected as it reflects the maximum comfortable walking distance from residences to basic facilities for older adults, while the 200-m buffer was considered because it represents a short walking distance (less than 5 min) for older adults experiencing some functional decline (Cerin et al., 2013). The accessibility of four types of neighborhood facilities was calculated by the number of each type of facility within the 200-m and 500-m buffers using the 2014 GeoCommunity Database (including exact location points of various types of facilities) provided by the Lands Department of Hong Kong government. Community facilities refer to welfare centers, community centers, and family service centers. Active leisure facilities refer to indoor sports venues and sports grounds. Passive leisure facilities refer to parks, pavilions, and open spaces. Public transportation refers to bus termini and subway stations. We focused on these specific neighborhood facilities because they are relevant to promoting SC and mental well-being among older adults as identified by previous literature (Barnett et al., 2018).

### Sociodemographic Characteristics

Sociodemographic characteristics included age (years), gender, marital status (married vs single/widowed/divorced/separated), education (no formal education, primary education, secondary education and above), and subjective financial sufficiency (measured by a 5-point Likert scale from 1 = very insufficient to 5 = abundant), years of residency, and self-rated health (1 = good/excellent health, 0 = poor health).

### Data Analysis

Descriptive statistics were calculated for sample characteristics, perceived age-friendly environment, and the availability of neighborhood facilities within two buffer areas. The Pearson correlations between age-friendly environment, availability of neighborhood facilities, SC, and subjective well-being were calculated. The intraclass correlation coefficient for multilevel models was 0.017, indicating no evidence of significant clustering effects in the data (Hofmann et al., 2000). Then, we applied single-level structural equation modeling (SEM) to test the theoretical framework and hypotheses. First, two separated measurement models of SC and mental well-being were examined. Second, structural models were built with cognitive and structural SC mediating relationships between eight domains of perceived age-friendliness of the environment and the availability of facilities in the neighborhood and mental well-being. All control variables were regressed on the dependent variables and mediators. The direct, indirect, and total effects were obtained. A mean variance inflation factor of less than 2 indicated no evidence of multicollinearity. A range of measures, comparative fit index and Tucker–Lewis index > 0.90, root mean square error of approximation ≤ 0.08, and a standardized root mean square residual ≤ 0.08 were used to assess model fit (Hu & Bentler, 1999). Estimates with a *p* value < .05 (two-tailed) were interpreted as statistically significant. We used Mplus 8 software (Muthén & Muthén, 2017) for SEM and STATA for basic data analysis.

## Results

### Sample Characteristics

Table 1 presents the respondents’ characteristics. Their mean age was 73.98 (standard deviation, *SD* = ±7.70). Most respondents were female (78.9%), married (51.1%), with primary school level education or below (51.2%), and lived in their neighborhoods for over 30 years. Less than half had good self-rated health (41.95%) but reported financial sufficiency (3.04 ± 0.62). They had good affective-emotional and psychological-functioning well-being. The mean scores of “trust,” “reciprocity,” and “sense of community” ranged from 3.41 to 3.77, indicating respondents’ possession of relatively high cognitive SC. Respondents had more than one organizational membership, high levels of social participation, but low levels of volunteering. Respondents perceived neighborhood environment as age-friendly in all domains. Table 1 also presents the availability of neighborhood facilities within 200-m and 500-m buffer areas. The largest number of neighborhood facilities in 500-m buffer areas was passive leisure facilities, followed by active leisure facilities, transportation, and community centers. Similar patterns were found in a 200-m buffer area. Supplementary Table 2 shows the correlation among the study variables.

**Table 1.** Sample Characteristics (*N* = 1,277)

Variables	Mean (*SD*)	*N* (%)
Age (range = 60–99 years)	73.98 (7.7)	
Sex		
Female		1,008 (78.94)
Male		269 (21.06)
Marital status		
Married		653 (51.14)
Others		624 (48.86)
Educational level		
No formal education		195 (15.27)
Primary		459 (35.94)
Secondary and above		623 (48.79)
Residence years (range = 1–91)	34.89 (17.24)	
Self-rated good health		536 (41.97)
Feeling sufficiency about money (range = 1–5)	3.04 (0.62)	
Mental well-being (emotional; range = 1–5)		
Feeling optimistic	3.45 (0.99)	
Feeling useful	3.75 (1.01)	
Feeling relaxed	3.79 (0.94)	
Mental well-being (functional; range = 1–5)		
Dealing with problems well	3.74 (0.92)	
Thinking clearly	3.78 (0.93)	
Feeling close to other people	3.95 (0.93)	
Making up my own mind about things	3.92 (0.9)	
Cognitive social capital (range = 1–5)		
Trust (range = 1–5)	3.51 (0.61)	
Reciprocity (range = 1–5)	3.41 (0.78)	
Sense of community (range = 1–5)	3.77 (0.55)	
Structural social capital		
Organizational membership (range = 0–7)	1.84 (1.54)	
Social participation (range = 0–1)	0.74 (0.44)	
Volunteering (range = 0–1)	0.42 (0.49)	
Perception of age-friendly environment (range = 1–6)		
Outdoor space	4.18 (1.11)	
Transport	4.57 (0.92)	
Housing	4.16 (1.16)	
Social participation	4.42 (1.06)	
Respect	4.43 (1.07)	
Volunteer	4.48 (1.07)	
Information	4.35 (1.05)	
Community and health service	4.32 (1.09)	
Number of neighborhood facilities (200-m buffer)		
# of community facilities (range = 0–5)	0.79 (1.14)	
# of active leisure facilities (range = 0–11)	1.67 (2.38)	
# of passive leisure facilities (range = 0–26)	3.47 (4.07)	
# of transportation facilities (range = 0–19)	1.74 (2.75)	
The number of neighborhood facilities (500-m buffer)		
# of community facilities (range = 0–15)	4.53 (2.54)	
# of active leisure facilities (range = 0–60)	11.51 (5.35)	
# of passive leisure facilities (range = 0–66)	25.49 (11.72)	
# of transportation facilities (range = 0–25)	9.29 (4.95)	

*Notes*: *SD* = standard deviation; # = number.

### Measurement Models of SC and Mental Well-Being

Supplementary Table 3 shows that most of the fit indices in two separate measurement models of SC and subjective well-being reached or were close to the acceptable level. The factor loadings ranged from 0.64 to 0.76 for cognitive SC, 0.43 to 0.56 for structural SC, 0.66 to 0.80 for affective-emotional well-being, and 0.62 to 0.83 for psychological-functioning well-being. All factor loadings were statistically significant, indicating that observed indicators well represented all the latent constructs.

### Direct Associations Between Neighborhood Environment, SC, and Mental Well-Being

**Figure 2.**
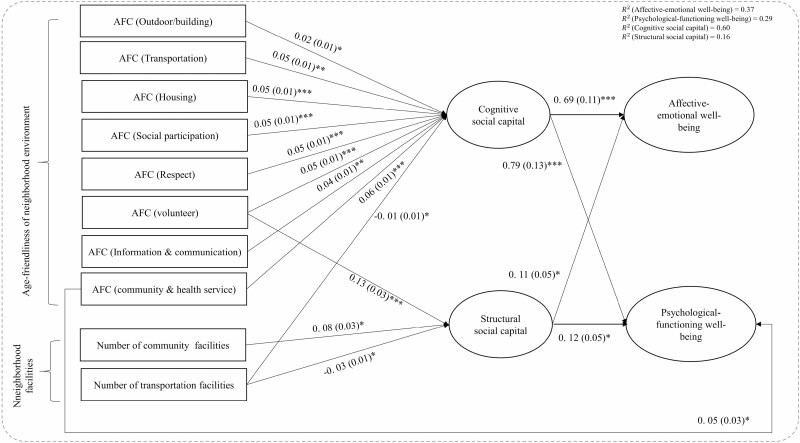
Unstandardized estimates of structural equation modeling (200-m buffer). *Notes*: AFC = age-friendly city. Model fit indices: χ^2^ = 784.176, *p* < .001, root mean square error of approximation = 0.043, comparative fit index = 0.920, Tucker–Lewis index = 0.882, standardized root mean square residual = 0.040. Results are controlled for age, gender, marital status, education, self-rated health, subjective financial status, and year of residence. Only statistically significant paths are shown (unstandardized estimates) and standard errors are displayed in parentheses. Correlated error values between cognitive and structural social capital, and correlated error values between feeling and functioning subjective well-being are not shown but are available upon request. ***p < .001, **p < .01, *p < .05.

Figure 2 shows the SEM analysis for the 200-m buffer area. Unstandardized coefficients for statistically significant pathways are presented. Three of the four fit indices fit the data well. Cognitive SC was associated with better affective-emotional (β = 0.69, *p* < .001) and psychological-functioning well-being (β = 0.79, *p* < .001). Structural SC was associated with better affective-emotional (β = 0.11, *p* < .05) and psychological-functioning well-being (β = 0.12, *p* < .05). We only found significant and direct associations between age-friendly neighborhood environment in domains of community and health services (β = 0.05, *p* < .05) and psychological-functioning well-being. All eight domains of perceived age-friendly environment were significantly associated with higher levels of cognitive SC. Only perceived age-friendly environment in the volunteering domain (β = 0.13, *p* < .001) was associated with higher levels of structural SC. The number of community centers was significantly associated with higher levels of structural SC (β = 0.08, *p* < .05). The number of transportation facilities was significantly associated with lower levels of cognitive (β = −0.01, *p* < .05) and structural SC (β = −0.03, *p* < .05), respectively.

**Figure 3.**
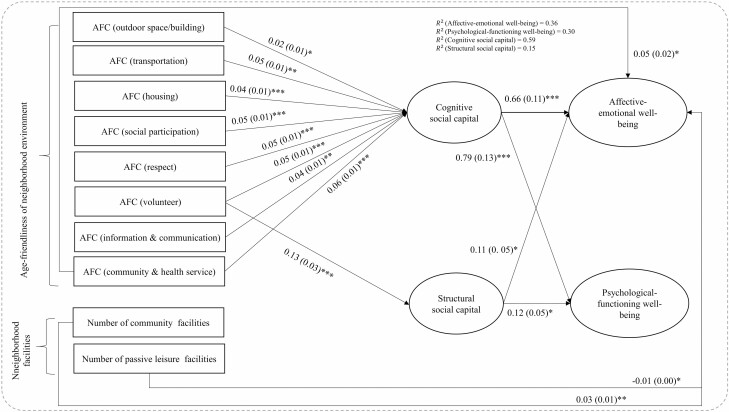
Unstandardized estimates of structural equation modeling (500-m buffer). *Notes*: AFC = age-friendly city. Model fit indices: χ^2^ = 760.13, *p* < .001, root mean square error of approximation = 0.042, comparative fit index = 0.921, Tucker–Lewis index = 0.886, standardized root mean square residual = 0.040. Results are controlled for age, gender, marital status, education, self-rated health, subjective financial status, and years of residence. Only statistically significant paths are shown (unstandardized estimates) and standard errors are displayed in parentheses. Correlated error values between cognitive and structural social capital, and correlated error values between feeling and functioning subjective well-being are not shown but are available upon request. ***p < .001, **p < .01, *p < .05.

Figure 3 shows results of SEM analysis for a 500-m buffer area. Unstandardized coefficients for statistically significant pathways are presented. Three of the four fit indices fit the data well. Most findings in SEM analysis from a 500-m buffer area were consistent with those from a 200-m buffer area, except for the associations between neighborhood facilities (community centers and passive leisure facilities) and cognitive/structural SC. Additionally, we found that affective-emotional well-being was positively and directly associated with community centers (β = 0.03, *p* < .01) but negatively associated with passive leisure facilities (β = −0.01, *p* < .05).

### Indirect and Total Associations Between Neighborhood Environment and Mental Well-Being

Table 2 summarizes indirect and total effects of neighborhood environment on mental well-being measured in 200-m buffer areas. Total indirect effects were equal to the sum of the indirect effects of neighborhood environment through cognitive and structural SC. Within a 200-m buffer, cognitive SC significantly mediated associations between affective-emotional well-being and age-friendly environment in the transportation (β = 0.04, *p* = .001), housing (β = 0.04, *p* < .001), and community and health service (β = 0.05, *p* < .001) domains by 67% (0.6 = 0.04/0.06), 75%, and 50%, respectively. The total indirect effect of perceived age-friendliness of neighborhood environment on affective-emotional well-being in outdoor spaces/buildings (β = 0.02, *p* < .05), social participation (β = 0.03, *p* < 0.01), respect (β = 0.04, *p* < .001), volunteering (β = 0.04, *p* < .001), and information/communication (β = 0.02, *p* < .01) on affective-emotional well-being were significant and mainly mediated by cognitive SC, although their total effects were not significant. Structural SC only significantly mediated associations between affective-emotional well-being and age-friendly environment in the volunteering domain (β = 0.01, *p* < .05). The total indirect effect of the availability of community centers was significant, although its total effect was not significant. The negative association between affective-emotional well-being and the number of transportation facilities was significantly mediated by cognitive SC (β = −0.01, *p* < .05).

**Table 2.** Unstandardized Estimates of Indirect and Total Effects of Neighborhood Environment on Mental Well-Being Through Social Capital (200-m Buffer)

200-m buffer	Indirect effects						Total effects	
	→ Cognitive SC		→ Structural SC		→ Cognitive + structural SC			
	β (*SE*)	*p* Value	β (*SE*)	*p* Value	β (*SE*)	*p* Value	β (*SE*)	*p* Value
Affective-emotional well-being								
Perception of age-friendly environment								
Outdoor space and building	0.02 (0.01)	.016	0 (0)	.870	0.02 (0.01)	.034	−0.01 (0.02)	.587
Transport	0.04 (0.01)	.001	0 (0)	.538	0.03 (0.01)	.004	0.06 (0.03)	.035
Housing	0.03 (0.01)	<.001	0 (0)	.341	0.03 (0.01)	.004	0.04 (0.02)	.049
Social participation	0.03 (0.01)	.002	0 (0)	.275	0.03 (0.01)	.001	0.05 (0.02)	.053
Respect	0.04 (0.01)	<.001	0.01 (0)	.116	0.04 (0.01)	<.001	0.04 (0.02)	.094
Volunteer	0.04 (0.01)	<.001	0.01 (0.01)	.040	0.05 (0.01)	<.001	0.02 (0.02)	.279
Information	0.02 (0.01)	.007	0.01 (0)	.184	0.03 (0.01)	.003	0 (0.02)	.857
Community and health service	0.04 (0.01)	<.001	0 (0)	.297	0.04 (0.01)	.001	0.08 (0.02)	<.001
The availability of neighborhood facilities								
Community facilities	0.01 (0.01)	.210	0.01 (0.01)	.079	0.02 (0.01)	.049	0.04 (0.02)	.091
Transportation facilities	−0.01 (0.001)	.015	0 (0)	.070	−0.01 (0)	.002	−0.01 (0.01)	.564
Psychological-functioning well-being								
Perception of age-friendly environment								
Outdoor space and building	0.02 (0.01)	.016	0 (0)	.870	0.02 (0.01)	.033	−0.02 (0.02)	.436
Transport	0.04 (0.01)	.001	0 (0)	.538	0.04 (0.01)	.004	0.03 (0.03)	.351
Housing	0.04 (0.01)	<.001	0 (0)	.343	0.03 (0.01)	.004	0.04 (0.02)	.071
Social participation	0.03 (0.01)	.002	0.01 (0)	.279	0.04 (0.01)	.001	0.05 (0.03)	.045
Respect	0.04 (0.01)	<.001	0.01 (0.01)	.121	0.05 (0.01)	<.001	0.01 (0.03)	.653
Volunteer	0.04 (0.01)	<.001	0.02 (0.01)	.044	0.06 (0.01)	<.001	0.04 (0.03)	.081
Information	0.03 (0.01)	.007	0.01 (0.01)	.187	0.03 (0.01)	.003	0 (0.03)	.904
Community and health service	0.05 (0.01)	<.001	0 (0)	.298	0.05 (0.01)	<.001	0.10 (0.03)	<.001
The availability of neighborhood facilities								
Community facilities	0.01 (0.01)	.210	0.01 (0.01)	.085	0.02 (0.01)	.051	0.05 (0.02)	.044
Transportation	−0.01 (0)	.015	0 (0)	.075	−0.01 (0)	.002	−0.01 (0.01)	.187

*Notes*: β = unstandardized estimated; *SE* = standard error. Results are controlled for age, gender, marital status, education, self-rated health, subjective financial status, and year of residence. Only statistically significant paths are shown. Other findings are available upon request.

We found similar patterns related to pathways in relationships between neighborhood environment and psychological-functioning well-being. Cognitive SC significantly mediated associations between psychological-functioning well-being and age-friendly environment in the social participation (β = 0.03, *p* < .01) and community/health service (β = 0.05, *p* < .001) domains by 60% and 50%, respectively. The total indirect effects of perceived age-friendliness of neighborhood environment on psychological-functioning well-being in the domains of outdoor space/building, transportation, housing, respect, volunteering, and information/communication were significant and mainly mediated by cognitive SC. Structural SC only significantly mediated associations between psychological-functioning well-being and age-friendly environment in the volunteering domain. The negative association between psychological-functioning well-being and the number of transportation facilities was significantly mediated by cognitive SC (β = −0.01, *p* < .05). Most findings related to the indirect associations between the perceived age-friendliness of neighborhood environment and mental well-being were similar for the 200-m and 500-m buffers (see Supplementary Table 4).

## Discussion

Our study contributes new knowledge to existing literature by substantiating cognitive and structural SC as key pathways in relationships between neighborhood environment and older adults’ mental well-being. The study generates several additional insights.

First, our findings revealed that higher levels of cognitive and structural SC are associated with better affective-emotional and psychological-functioning well-being among older people, further confirming the findings from previous studies (Ehsan & De Silva, 2015; Ehsan et al., 2019). It indicates that both cognitive and structural SC play important roles in maintaining older adults’ mental well-being.

Second, although we found direct relationships between neighborhood environment and mental well-being, they varied by neighborhood environmental attributes. Specifically, perceived age-friendly environment in community and health services was directly and positively associated with better mental well-being, consistent with previous studies (Lam et al., 2020). Accessible community and health services can encourage the use of preventive care and timely care support for older adults in need, promoting mental health. In addition, we found more community centers within a 500-m buffer area were directly associated with better affective-emotional well-being. More community centers within a 500-m buffer area can help older adults maintain a sense of being active, helping to improve their affective-emotional well-being (Levasseur et al., 2011; Lu et al., 2021). Unlike previous research, our study revealed that the number of passive leisure facilities (500-m buffer) was negatively and directly associated with older adults’ affective-emotional well-being. These findings contrast with previous studies in the West (Peace et al., 2005) but are consistent with the features of high-density Asian cities (Lu et al., 2021). Previous studies have found that passive leisure facilities, like parks and recreational places, can contribute to greater personal happiness, especially for older adults who generally cannot afford social activities (Peace et al., 2005). However, previous studies found that older adults in Hong Kong were dissatisfied with small and crowded parks (Chui et al., 2018; Lu et al., 2021). It is possible that a larger number of passive leisure facilities within a 500-m buffer could be too small to provide enough amenities (e.g., beaches) to fulfill older adults’ leisure needs. They may even be too small for older people to enjoy meaningful social exchange with other people.

Third, this study advances the theoretical understanding of the environment–mental health discourse by presenting evidence that both meaning-based and network-based processes mediate the relationship between neighborhood environment and older adults’ mental well-being. Previous studies only tested direct effects of perceived age-friendly environment on mental well-being in later life (Nieboer & Cramm, 2018). Eight domains of perceived age-friendliness neighborhood environment were significantly and indirectly associated with better affective-emotional and psychological-functioning well-being through a higher level of cognitive SC. These associations indicate that perceived age-friendliness neighborhood environment shape older adults’ sense of belonging, reciprocity, and trust, which in turn facilitate their achievement of affective-emotional and psychological-functioning well-being. Structural SC only mediated associations between perceived age-friendliness neighborhood environment in the volunteering domain and affective-emotional and psychological-functioning well-being. It indicates that opportunities for volunteering in the neighborhood are more influential than other domains of the age-friendly environment for encouraging older adults to maintain social connections (Cramm et al., 2018).

Fourth, we found that the number of transportation facilities within a 200-m buffer indirectly lowered affective-emotional and psychological-functioning well-being through lower levels of cognitive/structural SC. This contrasts with previous Western literature showing that the availability of public transportation is essential for older adults’ independence and mental well-being, especially when they become unable to drive (Shrestha et al., 2017). However, public transportation in Hong Kong maximizes the needs of the working population and efficiency, which may undermine the needs of older adults who travel during nonpeak hours (Chui et al., 2018). More public transit facilities within a 200-m buffer are likely to increase noise and traffic and trigger a sense of unsafety that may undermine older adults’ happiness and psychological functioning (Rambaldini-Gooding et al., 2021).

Fifth, our study revealed that the number of community centers in a 200-m buffer was significantly and indirectly associated with better affective-emotional well-being and psychological-functioning well-being (marginally), with structural SC being the key mediator. Previous studies have found that more community centers can encourage more social networks, interaction, and social participation among older adults because older adults find it convenient to visit community centers in their neighborhoods and maintain their structural SC (Lu et al., 2021).

Our study expands the existing knowledge by revealing that the meaning-based process played a more important role in facilitating older adults’ mental well-being as more significant mediating effects of cognitive SC were found. It is possible that network-based processes (e.g., the mediating role of structural SC) decrease in importance for aging well when people move into advanced age with varying levels of functional limitations. Older people tend to develop a strong sense of belonging to their neighborhood, trust in their community members, attachment to the place and the neighbors surrounding them (Wahl et al., 2012).

In addition to direct and indirect associations between neighborhood environment and mental well-being, we found discrepancies between two observed areas. For instance, more mediating effects of SC between neighborhood facilities and mental well-being were observed in a 200-m buffer area, while more direct effects of neighborhood facilities were found in a 500-m buffer area. Taking public transportation as an example, a greater number of public transportation facilities located within a 200-m buffer may prohibit social interaction. In comparison, a greater number of public transportation facilities within a 500-m buffer area may potentially threaten older adults’ feelings and mental well-being. Future studies can investigate how different buffer areas shape the opportunities related to older adults’ feelings and actions to maintain their mental well-being.

Our study has several strengths. It is theoretically informed, integrating both ETA and SC theories, and offers a new theoretical model to explain relationships between neighborhood environment and mental health in old age. The study included a large sample in a large urban and high-density Asian city, applied both subjective and objective attributes of neighborhood environment, estimated the mediating roles of cognitive and structural SC on the two dimensions of affective-emotional and psychosocial-functioning well-being simultaneously using two buffers, and applying SEM analysis. Our study also has some limitations. First, the cross-sectional nature of the data does not allow us to draw causal associations between neighborhood environment and mental well-being. Second, the study did not draw on a random sample. Thus, our findings may not be generalizable to other populations of older people (e.g., those with disabilities). Future studies may consider including older adults with and without disabilities. Third, we only included individual-level SC without considering multilevel SC and its various types (bonding, linking, and bridging). Future studies that apply both individual and multilevel measures of SC and various forms of SC to capture comprehensive SC profiles are needed to better understand the effects of neighborhood environment on mental well-being. Last, the respondents in our sample have a variety of residence years, ranging from 1 to 91. The mediating effects of SC in the model may differ in older adults who have lived in the community for a long time versus those who are newcomers. Future studies should further investigate whether residence years moderate the mediating effects of SC in the relationship between neighborhood environment and well-being among older adults.

Our findings can provide practical implications for urban planning strategies and aging-in-place policies. First, we highlight the role of an age-friendly environment in improving better mental well-being among older adults. Pre-coronavirus disease 2019 (COVID-19) research has documented the positive effects of an age-friendly environment on older adults’ mental health (Au et al., 2020). Our study was conducted during the COVID-19 pandemic and documented the persistence of such protective roles. Indeed, a recent study has found that an age-friendly environment (e.g., communication and information domain) mitigated older adults’ infodemic-related anxiety during COVID-19 (Wong et al., 2022). Thus, policy efforts to create a more age-friendly neighborhood environment should continue in highly urbanized cities with rapidly aging populations to enhance SC for better mental well-being, even during the pandemic. Second, we revealed that a meaning-based process outweighed a network-based process, indicating the need for strategic planning to improve cognitive and structural SC for better mental well-being in the aging society. Policymakers and social workers should create an age-friendly environment providing more volunteering opportunities and community centers to enhance structural SC, further encouraging better mental well-being among older adults. Last, we identified the negative effects of the number of transportation facilities and passive leisure activities on mental well-being and SC. This implies that the quality of these facilities could be more important than their quantity in terms of affecting older adults’ cognitive SC and mental well-being. Therefore, identifying the key aspects of quality to satisfy older adults might be a more effective way of improving their mental well-being.

## Conclusion

Our study innovatively applied an integrative theoretical framework to examine the mediating effects of cognitive and structural SC in relationships between perceived age-friendly neighborhood environment and subjective measures of neighborhood facilities and mental well-being. Both meaning-based and network-based processes were key pathways between neighborhood environment and mental well-being. We expanded the ecological model of aging by linking it to SC theory, thus providing evidence of the importance of an age-friendly environment in enhancing SC and, thus, mental well-being in later life. In Asian societies with rapidly aging populations, it is imperative to improve neighborhood age-friendliness and ensure the availability of community centers to optimize large-scale mental well-being improvement strategies.

## Supplementary Material

igac070_suppl_Supplementary_Material
