# Supplementary material for: Neighborhood Environment and Mental Well-Being Among Chinese Older Adults: The Mediating Role of Social Capital
Source: Innov Aging. 2022 Nov 17;6(7):igac070. doi: 10.1093/geroni/igac070 (PMC9799048; doi:10.1093/geroni/igac070)
Supplement: igac070_suppl_Supplementary_Material [file igac070_suppl_supplementary_material.docx]

Supplementary Table 1. A summary of the measurements used for constructing social capital in this study

| **Cognitive social capital** | | |
| --- | --- | --- |
| Subconstructs | Measurements | Reference |
| Trust | Six-item Generalized Trust Scale was used to assess participants’ beliefs about trustworthiness of others living in the neighborhood. It is based on a 5-point Likert scale (1= completely disagree to 5= completely agree). | Yamagishi et al., 1994 |
| Reciprocity | Two questions were used to assess reciprocity, “Do you think residents are concerned about issues that not only relate to themselves, but also others?” and “Most residents living in this community could help each other.” Responses were collected on a 5-point Likert scale (1= completely disagree to 5= completely agree). | Originally developed by De Silva et al. (2006) and translated and applied into Chinese society by (Lu et al., 2020) |
| Sense of belonging | Eight-item Brief Sense of Community Scale (BSCS), which consists of four dimensions, including needs fulfilment, group membership, influence, and emotional connection and uses a 5-point Likert-type scale (1=strongly disagree to 5=strongly agree). | Peterson et al., 2008 |
| **Structural social capital** | | |
| Organisational membership | Participants were asked if they held *organizational membership* (0=No, 1=Yes) in the following list of seven categorisations of organizations and culturally adapted to Hong Kong by the researchers: (1) self-initiated activity groups (e.g., Tai chi), (2) cultural/entertainment/sports-related groups (e.g., sports clubs), (3) religious groups, (4) charitable organizations, (5) political parties, (6) labor unions, and (7) social organizations (e.g., women’s groups). | Chen et al., 2009; De Silva et al., 2006 |
| Social participation | Participants were asked if they had *participated* (0=No, 1=Yes) in social activities or utilized the social services provided by centres for older people in the past three months. | De Silva et al., 2006; Lu et al., 2020 |
| Volunteering | Participants were asked whether they volunteered (0=No, 1=Yes) in the past three months. | De Silva et al., 2006; Lu et al., 2020 |

Notes: All the references shown in this table have been listed in the reference list in the main text.

Supplementary Table 2. Correlation (N= 1,277)

| # | Variables | 1 | 2 | 3 | 4 | 5 | 6 | 7 | 8 | 9 | 10 | 11 | 12 | 13 |
| --- | --- | --- | --- | --- | --- | --- | --- | --- | --- | --- | --- | --- | --- | --- |
| 1 | SWEMWBS Item 1 | 1 |  |  |  |  |  |  |  |  |  |  |  |  |
| 2 | SWEMWBS Item 2 | 0.52*** | 1 |  |  |  |  |  |  |  |  |  |  |  |
| 3 | SWEMWBS Item 3 | 0.55*** | 0.57*** | 1 |  |  |  |  |  |  |  |  |  |  |
| 4 | SWEMWBS Item 4 | 0.44*** | 0.56*** | 0.60*** | 1 |  |  |  |  |  |  |  |  |  |
| 5 | SWEMWBS Item 5 | 0.35*** | 0.47*** | 0.51*** | 0.62*** | 1 |  |  |  |  |  |  |  |  |
| 6 | SWEMWBS Item 6 | 0.38*** | 0.39*** | 0.50*** | 0.47*** | 0.43*** | 1 |  |  |  |  |  |  |  |
| 7 | SWEMWBS Item 7 | 0.38*** | 0.50*** | 0.47*** | 0.64*** | 0.59*** | 0.52*** | 1 |  |  |  |  |  |  |
| 8 | Trust | 0.25*** | 0.18*** | 0.25*** | 0.20*** | 0.19*** | 0.31*** | 0.20*** | 1 |  |  |  |  |  |
| 9 | Reciprocity | 0.24*** | 0.17*** | 0.24*** | 0.16*** | 0.11*** | 0.31*** | 0.15*** | 0.55*** | 1 |  |  |  |  |
| 10 | Sense of community | 0.33*** | 0.26*** | 0.33*** | 0.30*** | 0.21*** | 0.34*** | 0.24*** | 0.46*** | 0.48*** | 1 |  |  |  |
| 11 | Organizational membership | 0.11*** | 0.12*** | 0.12*** | 0.13*** | 0.10*** | 0.16*** | 0.13*** | 0.15*** | 0.20*** | 0.24*** | 1 |  |  |
| 12 | Social participation | 0.03 | 0.07* | 0.05 | 0.04 | 0.05 | 0.07* | 0.03 | 0.06* | 0.05 | 0.08** | 0.22*** | 1 |  |
| 13 | Volunteering | 0.08** | 0.15*** | 0.12*** | 0.12*** | 0.11*** | 0.1*** | 0.09*** | 0.05 | 0.07* | 0.09*** | 0.19*** | 0.33*** | 1 |
|  | Perceived Age-friendly environment | | |  |  |  |  |  |  |  |  |  |  |  |
| 14 | AFE1 | 0.17*** | 0.14*** | 0.18*** | 0.14*** | 0.09** | 0.16*** | 0.10*** | 0.29*** | 0.29*** | 0.36*** | 0.09** | 0.01 | 0.02 |
| 15 | AFE2 | 0.25*** | 0.17*** | 0.22*** | 0.17*** | 0.10*** | 0.22*** | 0.14*** | 0.36*** | 0.33*** | 0.43*** | 0.09** | 0.02 | 0.03 |
| 16 | AFE3 | 0.22*** | 0.15*** | 0.23*** | 0.18*** | 0.09** | 0.24*** | 0.14*** | 0.32*** | 0.36*** | 0.44*** | 0.09** | 0.04 | 0.01 |
| 17 | AFE4 | 0.20*** | 0.14*** | 0.23*** | 0.17*** | 0.09** | 0.19*** | 0.16*** | 0.30*** | 0.36*** | 0.47*** | 0.16*** | 0.11*** | 0.06* |
| 18 | AFE5 | 0.21*** | 0.13*** | 0.20*** | 0.14*** | 0.08** | 0.19*** | 0.09** | 0.32*** | 0.34*** | 0.42*** | 0.15*** | 0.08** | 0.09** |
| 19 | AFE6 | 0.16*** | 0.16*** | 0.19*** | 0.17*** | 0.12*** | 0.18*** | 0.13*** | 0.27*** | 0.32*** | 0.43*** | 0.17*** | 0.13*** | 0.15*** |
| 20 | AFE7 | 0.18*** | 0.17*** | 0.20*** | 0.17*** | 0.10*** | 0.18*** | 0.13*** | 0.32*** | 0.31*** | 0.46*** | 0.12*** | 0.1*** | 0.07* |
| 21 | AFE8 | 0.25*** | 0.22*** | 0.23*** | 0.23*** | 0.12*** | 0.21*** | 0.18*** | 0.30*** | 0.34*** | 0.50*** | 0.13*** | 0.03 | 0.01 |
|  | The number of neighborhood facilities (within a 200-meter buffer) | | | | | |  |  |  |  |  |  |  |  |
| 22 | Community facilities | 0.09** | 0.03 | 0.06* | 0.03 | 0.03 | -0.02 | 0.02 | 0.055* | .081** | 0.05 | 0.04 | 0.04 | 0.01 |
| 23 | Leisure facilities (A) | -0.01 | -0.01 | 0.01 | 0.00 | -0.01 | -0.04 | -0.02 | 0.03 | 0.03 | 0.01 | -0.01 | 0.04 | 0.01 |
| 24 | Leisure facilities (P) | .072* | 0.02 | 0.03 | -0.04 | -0.02 | -0.03 | -0.02 | 0.00 | 0.02 | 0.01 | -0.04 | -0.01 | 0.02 |
| 25 | Transportation facilities | 0.02 | 0.01 | 0.02 | -0.03 | 0.02 | -0.05 | -0.04 | -0.05 | 0.00 | -0.05 | -0.04 | -0.07 | -0.01 |
|  | The number of neighborhood facilities (within a 500-meter buffer) | | | | | |  |  |  |  |  |  |  |  |
| 26 | Community facilities | 0.04 | -0.01 | -0.01 | -0.02 | -0.02 | -0.07* | -0.03 | 0.01 | 0 | -0.02 | -0.03 | -0.06 | -0.01 |
| 27 | Leisure facilities (A) | -0.05 | -0.04 | -0.05 | -0.04 | -0.03 | -0.08** | -0.05 | -0.02 | -0.04 | -0.04 | -0.02 | -0.05 | -0.02 |
| 28 | Leisure facilities (P) | -0.03 | -0.06* | -0.05 | -0.07 | -0.07 | -0.07* | -0.06* | 0 | -0.01 | -0.02 | -0.05 | -0.07 | -0.02 |
| 29 | Transportation facilities | -0.01 | 0.00 | -0.04 | -0.01 | 0.02 | -0.08** | -0.03 | -0.01 | -0.06* | -0.01 | -0.04 | -0.07 | 0.01 |

Notes: SWEMWBS Item 1-7 = sub-items of Warwick-Edinburgh Mental Well-being Scale (SWEMWBS), including “feeling optimistic”, “feeling useful”, “feeling relaxed”, “dealing with problems well”, “thinking clearly”, “feeling close to other people”, and “make up my own mind about things”. AFE1-8 = Age friendly environment, including outdoor space, transportation, housing, social participation, respect, volunteering, information, and community & health service. A = active. P = Passive. ***p < .001, **p < .01, *p < .05.Supplementary Table 3. Measurement models (N= 1,277)

| **(1) Measurement model for social capital (N=1,277)** | | |  |  |  |
| --- | --- | --- | --- | --- | --- |
| *A. Estimates of factor loading* | |  |  |  |  |
| Latent variables | Measurement variables | Standardized estimates | SE | p-value | Fit statistics |
| Cognitive social capital | Trust | 0.72 | 0.02 | <0.001 | Chi-Square = 67.51, df = 8, p < 0.001, RMSEA = 0.076, CFI = 0.950, TLI = 0.907, SRMR = 0.050 |
|  | Reciprocity | 0.76 | 0.02 | <0.001 |  |
|  | Sense of community | 0.64 | 0.02 | <0.001 |  |
| Structural social capital | Organizational membership | 0.43 | 0.04 | <0.001 |  |
|  | Social participation | 0.56 | 0.04 | <0.001 |  |
|  | Volunteering | 0.52 | 0.04 | <0.001 |  |
| *B. Covariances of two latent variables* | |  |  |  |  |
| Cognitive social capital | Structural social capital | 0.28 | 0.05 | <0.001 |  |
|  |  |  |  |  |  |
| **(2) Measurement model for mental well-being (N=1,277)** | | | |  |  |
| *A. Estimates of factor loading* | |  |  |  |  |
| Latent variables | Measurement variables | Standardized estimates | SE | p-value | Fit statistics |
| Affective-emotional well-being | Feeling optimistic | 0.66 | 0.02 | <0.001 | Chi-Square = 133.85, df = 13, p < 0.001, RMSEA = 0.085, CFI = 0.969, TLI = 0.95, SRMR = 0.028 |
|  | Feeling useful | 0.75 | 0.02 | <0.001 |  |
|  | Feeling relaxed | 0.80 | 0.02 | <0.001 |  |
| Psychological-functioning well-being | Dealing with problems well | 0.83 | 0.01 | <0.001 |  |
|  | Thinking clearly | 0.74 | 0.02 | <0.001 |  |
|  | Feeling close to other people | 0.62 | 0.02 | <0.001 |  |
|  | Make up my own mind about things | 0.77 | 0.01 | <0.001 |  |
| *B. Covariances of two latent variables* | |  |  |  |  |
| Affective-emotional well-being | Psychological-functioning well-being | 0.85 | 0.02 | <0.001 |  |

Notes: SE = Standard error, df = degree of freedom, RMSEA = root mean square error of approximation, CFI = comparative fit index, TLI = Tucker–Lewis index, SRMR=standardized root-mean-square residual. Supplementary Table 4. Unstandardized Estimates of Indirect and Total Effects of neighborhood environment on mental well-being through social capital (500-meter buffer)

| 500-meter buffer | Indirect effects | | | | | | Total effects | |
| --- | --- | --- | --- | --- | --- | --- | --- | --- |
|  | → Cognitive SC | | → Structural SC | | → Cognitive + Structural SC | |  |  |
|  | β (S.E.) | p-value | β (S.E.) | p-value | β (S.E.) | p-value | β (S.E.) | p-value |
| **Affective-emotional well-being** | | |  |  |  |  |  |  |
| Perception of age-friendly environment | | |  |  |  |  |  |  |
| Outdoor space & building | 0.02 (0.01) | 0.045 | 0 (0) | 0.759 | 0.01 (0.01) | 0.094 | -0.01 (0.02) | 0.548 |
| Transport | 0.03 (0.01) | 0.002 | 0 (0) | 0.611 | 0.03 (0.01) | 0.011 | 0.06 (0.03) | 0.032 |
| Housing | 0.03 (0.01) | 0.001 | 0 (0) | 0.689 | 0.03 (0.01) | 0.003 | 0.04 (0.02) | 0.042 |
| Social participation | 0.03 (0.01) | 0.001 | 0.01 (0) | 0.230 | 0.04 (0.01) | <0.001 | 0.05 (0.02) | 0.028 |
| Respect | 0.03 (0.01) | <0.001 | 0.01 (0) | 0.140 | 0.04 (0.01) | <0.001 | 0.03 (0.02) | 0.139 |
| Volunteer | 0.04 (0.01) | <0.001 | 0.01 (0.01) | 0.043 | 0.05 (0.01) | <0.001 | 0.02 (0.02) | 0.304 |
| Information | 0.02 (0.01) | 0.006 | 0 (0) | 0.263 | 0.03 (0.01) | 0.003 | 0 (0.02) | 0.939 |
| Community and health service | 0.04 (0.01) | <0.001 | 0 (0) | 0.260 | 0.04 (0.01) | 0.001 | 0.08 (0.02) | <0.001 |
| *The availability of neighborhood facilities* | |  |  |  |  |  |  |  |
| Community facilities | 0.01 (0) | 0.181 | 0 (0) | 0.985 | 0.01 (0) | 0.231 | 0.04 (0.01) | 0.002 |
| Passive leisure facilities | 0 (0) | 0.768 | 0 (0) | 0.240 | 0 (0) | 0.446 | -0.01 (0) | 0.025 |
| **Psychological-functioning well-being** | | |  |  |  |  |  |  |
| Perception of age-friendly environment | |  |  |  |  |  |  |  |
| Outdoor space & building | 0.02 (0.01) | 0.044 | 0 (0) | 0.759 | 0.02 (0.01) | 0.087 | -0.02 (0.02) | 0.376 |
| Transport | 0.04 (0.01) | 0.002 | 0 (0) | 0.612 | 0.04 (0.01) | 0.009 | 0.03 (0.03) | 0.367 |
| Housing | 0.03 (0.01) | 0.001 | 0 (0) | 0.689 | 0.03 (0.01) | 0.003 | 0.05 (0.02) | 0.056 |
| Social participation | 0.04 (0.01) | 0.001 | 0.01 (0) | 0.235 | 0.05 (0.01) | <0.001 | 0.06 (0.03) | 0.028 |
| Respect | 0.04 (0.01) | <0.001 | 0.01 (0.01) | 0.146 | 0.05 (0.01) | <0.001 | 0.01 (0.03) | 0.763 |
| Volunteer | 0.04 (0.01) | <0.001 | 0.02 (0.01) | 0.048 | 0.06 (0.01) | <0.001 | 0.04 (0.02) | 0.090 |
| Information | 0.03 (0.01) | 0.006 | 0.01 (0) | 0.267 | 0.03 (0.01) | 0.003 | 0 (0.03) | 0.895 |
| Community and health service | 0.05 (0.01) | <0.001 | -0.01 (0) | 0.263 | 0.04 (0.01) | 0.001 | 0.09 (0.03) | <0.001 |

Notes: β = unstandardized estimated. S.E. = Standard Error; Results are controlled for age, gender, marital status, education, self-rated health, subjective financial status, and year of residence. Only statistically significant paths are shown. Other findings are available upon request.
